# Translation, reliability and validity of the Turkish versions of Norwich Patellar Instability score and The Banff Patellar Instability Instrument 2.0

**DOI:** 10.1186/s13018-024-04612-3

**Published:** 2024-02-14

**Authors:** Engin Turkay Yilmaz, Ibrahim Mehmet Goymen, Melih Oral, Ozan Tuncay, Gokay Dursun, Egemen Turhan, Ahmet Mazhar Tokgozoglu

**Affiliations:** Orthopedics and Traumatology Department, Hacettepe University Medical School, Ankara, Turkey

## Abstract

**Background:**

Current treatment options for patellofemoral (PF) instability have required functional scoring systems. The Banff Patellar Instability Instrument (BPII) 2.0 and Norwich Patellar Instability (NPI) scores were explicitly created to meet the need to evaluate PF instability. Different patient-reported outcome measurements (PROMs) are used to evaluate anterior knee problems.

**Objectives:**

To test the validity and reliability of the Turkish version of the BPII 2.0 and NPI score.

**Study design and methods:**

Fifty-one patients that operated for PF instability, older than eighteen years old, were included in this study. Turkish translation of the BPII 2.0 and NPI scores was undertaken through translation into Turkish by an independent translator. Two tests were repeated seven days apart. Intraclass correlation coefficient (ICC) was used for test–retest reliability. Internal consistency was analyzed using Cronbach's alpha. Validity was assessed by correlating the Kujala and Lysholm knee scores.

**Results:**

Fifty-one patients (34 females/17 males), the average age was 25 ± 7, were included in this study. Cronbach's alpha value was 0.829 for BPII 2.0 and 0.843 for NPI for the first time answered by patients. ICC values applied to evaluate test–retest reliability were 0.904 (*p* < 0.05) for BPII 2.0 and 0.915 (*p* < 0.05) for NPI. There was a moderate correlation between the BPII 2.0 Turkish version and the Kujala score. There was a very high correlation between the Turkish version of the BPII 2.0 and Lysholm knee scores. An excellent negative correlation was found between Norwich and Kujala scores (*r* = −0.819, *p* < 0.05). The correlation coefficient between Norwich and Lysholm scores was −0.662, indicating a high negative correlation (*p* < 0.05). The correlation coefficients between the Turkish version of BPII 2.0 and NPI were −0.533 (*p* < 0.05).

**Conclusions:**

The Turkish version of the BPII 2.0 and NPI score is a reliable and valid instrument for Turkish-speaking patients with patellofemoral instability.

**Supplementary Information:**

The online version contains supplementary material available at 10.1186/s13018-024-04612-3.

## Introduction

Patellofemoral instability (PFI), especially in recent years, has begun to be more widely examined by surgeons, and more emphasis has been placed on its diagnosis and treatments [1]. Patellofemoral instability includes patellar dislocation and subluxation, and trochlear dysplasia, patella alta, and bony malalignment are the primary anatomic abnormalities of cause [2]. It is expected that its prevalence will increase due to the increasing youth population and sport activities.

New treatment options are being developed and researched by surgeons [3–7]. As a result of new developments in treatment, it has required the use of scoring systems for the evaluation of the functional results of patients [8]. Patient-reported outcome measurements (PROMs) are valuable instruments for evaluating the success of the surgery and patient satisfaction. PROMs used before were used to evaluate patellofemoral pain syndrome in which anterior knee pain was in the foreground rather than specifically questioning patellofemoral instability [9]. Lysholm Knee Scoring Scale, Kujala Score, Fulkerson Patellofemoral Score, and the International Knee Documentation Committee form are generally used to assess knee disorders [10–13]. Kujala and Lysholm knee scores were translated and validated into Turkish and have been used [14, 15].

With the need to investigate mainly the patellofemoral instability, Banff Patella Instability Instrument 2.0 and Norwich Patellar Instability score were published and started to be used [16–18]. To evaluate the functional results in future studies in Turkish-speaking communities, it was necessary to translate these two tests and make cultural adaptations.

We aimed to translate and culturally adapt BPII version 2.0 and NPI to Turkish and evaluate of reliability and validity of both scores, comparing them to Turkish versions of the Kujala score and the Lysholm Knee Scoring Scale.

## Patients and method

### PROMs

BPII was first released in 2013 by Hiemstra based on Anterior Cruciate Ligament–Quality of Life (ACL–QOL) with 32 questions [17]. BPII was revisited in 2016 with fewer questions after factor analysis [18]. This instrument contains five main topics, including symptoms/physical complaints, recreational activity work-related concerns and sport participation/competition, social/emotional, and lifestyle. Patients mark their answers with a slash on a 100-mm line. All of the questions have equal weight, and calculation is made by taking the average point of all answered questions, and a higher score means the patient has a better quality of life. BPII version 2.0 is translated and culturally adapted to German, Brazilian Portuguese, Dutch, Indonesian, Norwegian and Swedish [19–24].

In 2014, Smith et al. published NPI score for evaluating disease-specific outcomes for PF instability [16]. This scoring consists of 19 questions with a 5-grade Likert scale from "never" to "always" that reveal the patellofemoral symptoms of patients. With NPI score, patients are questioned about how often patellofemoral complaints and symptoms occur when patients move on different surfaces and in different directions, both at high and low activities. NPI scoring has a more complex scoring compared to others. The questions have different points, and the points obtained from the answered questions are added up and divided by the maximum points that can be obtained from the questions, and the percentage is taken. Higher scores from NPI show PF instability is more restricting for the patient, indicating a poor result. NPI was translated and adapted to Dutch and Brazilian Portuguese [21, 25].

Kujala score was published in 1993 for evaluating patellofemoral disorders [11]. Kujala has 13 questions for assessing anterior knee problems. This scoring is intended to evaluate anterior knee problems, and only one question specifically considers patellofemoral instability. All questions weigh the same point; the final score is between 0 and 100. Better final points mean better anterior knee function. Kujala score was translated to Turkish and successfully validated in 2018 [14].

Lysholm knee scale was first published in 1982, consisting of 8 questions with ease for patients and a shorter time for answering generally between other knee scales [10]. Although the Lysholm knee scale is designed to evaluate knees that have suffered ligament injuries as the primary purpose, it is also used for patellofemoral syndrome, patellar tendonitis, meniscus injuries, and other similar knee problems [26]. This scale, in which symptoms such as swelling and pain are questioned, is scored out of 100; the higher results mean better function. The Lysholm knee scoring scale was translated and validated into Turkish in 2013 by Celik et al. [15].

### Translation

The International Society carried out the translation process for Pharmacoeconomics and Outcome Research (ISPOR) guidelines [27]. NPI and BPII 2.0 are translated to Turkish separately by a bilingual orthopedic surgeon and electronics engineer. A committee evaluated both translations and turned them into one advanced translation for both scores. Translated scores were back-translated to English by another bilingual orthopedic surgeon and teacher. The committee reviewed these back translations and finalized the translations. Tests were applied to 15 non-patient people to determine their intelligibility, and the parts that people had difficulty understanding were noted, and appropriate changes were made (Additional files 1 and 2).

### Patients and application

Permission was obtained from Hacettepe University Ethics Committee. Patients who were operated on due to patellofemoral instability between December 2016–2020 were scanned from the archive, and 84 patients that operated for patellofemoral instability were listed. Inclusion criteria were: (1) being older than 18 years old, (2) being operated on due to patellar instability diagnosed by clinical examination and imaging, (3) being able to answer the questions on tests electronically by themselves. All four surveys were conducted face-to-face. Then, the patients were asked to answer the BPII 2.0 and NPI scores again after 7–10 days.

### Statistics

All statistical data calculations were made with SPSS version 23.0. Descriptive data were calculated using mean, standard deviation, and range. Normality was assessed using the Shapiro–Wilk test, and normal distribution was obtained. For construct validity of translated versions of BPII and NPII, both were compared to Turkish versions of Kujala and Lysholm Knee Scores to determine Pearson correlation coefficients. Acceptable values for correlation considered as higher than 0.4 (*r* = 0.00–0.20 poor, 0.21–0.40 fair, 0.41–0.60 good, 0.61–0.80 very good, 0.81–1.00 excellent) [28]. For determining internal consistency, Cronbach alpha was used and values between 0.70 and 0.95 were accepted adequate. Intraclass correlation coefficient (ICC) was used for test–retest reliability. A value above 0.4 was considered sufficient (*r* = 0.00–0.20 poor, 0.21–0.40 fair, 0.41–0.60 good, 0.61–0.80 very good, 0.81–1.00 excellent) [29]. We preferred 7–10 days between the two tests because we accepted that there would be no changes in the patient's condition related to their knees during this period. We confirmed that the patients received no additional treatment during this interval. The 15-percent limit accepted in previous translation studies was applied to evaluate ceiling and floor effects [19, 30]. If the frequency of patients who get the minimum and maximum scores that can be obtained in the questionnaires exceeds 15 percent, then ceiling and floor effects can be mentioned.

## Results

Finalized tests were performed on 51 patients (34 females /17 males) who were accepted to be included in this study. All patients could complete questionnaires the second time in 7–10 days. It was ensured that there was no change in the medical conditions of the patients during this period regarding their knees. The median age of the patients was 19 years (min 18–max 32).

### Reliability

Both Cronbach's alpha values were sufficient. Examination of the internal consistency of both questionnaires, Cronbach's alpha value was found to be 0.829 for BPII 2.0 and 0.843 for NPI for the first time answered by patients. ICC values applied to evaluate test–retest reliability were 0.904 (*p* < 0.05) for BPII and 0.915 (*p* < 0.05) for NPI.

### Descriptives

Patients scored an average of 62.725 ± 18.394 on the first completion and 64.706 ± 20.129 on the second completion of the BPII 2.0 Turkish Version. For NPI, these scores were 40.175 ± 24.305 and 38.380 ± 24.912 for the first and second completion, respectively. The results are shown in Table 1.

**Table 1:** Mean values and comparisons at first and second completions of NPI and BPII

	First completion		Second completion		n	t	p
	Mean	SD	Mean	SD			
Banff	62.725	18.394	64.706	20.129	51	− 1.241	0.220
Norwich	40.175	24.305	38.380	24.912	51	0.929	0.357

### Construct validity

A moderate correlation was found between the BPII 2.0 Turkish version and Kujala, with a coefficient of 0.529 (*p* < 0.05). There was a very high correlation between the BPII Turkish version and the Turkish version of the Lysholm knee score (*r* = 0.807, *p* < 0.05). Excellent negative correlation was found between Norwich and Kujala scores (*r* = −0.819, *p* < 0.05). The correlation coefficient between Norwich and Lysholm was −0.662, indicating a high negative correlation (*p* < 0.05). The correlation coefficients between the Turkish version of BPII 2.0 and NPI were −0.533 (*p* < 0.05). The results are shown in Table 2.

**Table 2:** Correlation of BPII and NPI with other scoring systems

	Kujala score	Lysholm score	BPII 2.0
BPII 2.0			
*r*	0.529	0.807	1
*p*	0.000	0.000	
NPI			
*r*	−0.819	−0.662	−0.553
*p*	0.000	0.000	0.000

### Floor and ceiling effects

No patients scored minimum and maximum scores in BPII 2.0 for the first time of completion (min = 28, max = 89) and the second time of completion (min = 16, max = 93). For the first time of completion, no patient had a minimum score, but one patient had a maximum score of 100 (min = 3, max = 100). For the second time of completion of NPI, only one patient had 100 points (min = 4, max = 100). When these results were evaluated, it was seen that the total ratio of patients who exceeded the minimum and maximum value did not exceed 15 percent, and based on this, it was seen that there was no floor and ceiling effect.

## Discussion

BPII 2.0 and Norwich's scales are more disease-specific than previously used measurements for PFI. These PROMs aimed to let surgeons more precisely evaluate the preoperative and postoperative functions of patients with patellofemoral instability. Adaptations of these PROMs to other languages and cultures have been made in the literature, and their reliability and validity have been tested. We have performed the translation and cultural adaptation of these PROMS in the Turkish-speaking population and demonstrated that they have adequate reliability and validity. Cronbach's alpha value to evaluate internal consistency was sufficient for the Turkish version BPII 2.0 and NPI (0.829 and 0.843, respectively). In the original studies, Banff Cronbach's alpha value was 0.91 for BPII 2.0 and 0.93 for NPI, comparable to our study's values [16, 18]. In the translation and reliability studies conducted for BPII 2.0 in other languages, these values were found to be 0.93 and 0.95 for the German version, 0.967 for the Dutch version, 0.97 for the Indonesian version, 0.97 for the Swedish version and 0.95 for the Norwegian version [19, 21–24]. Dutch translation of NPI had Cronbach's alpha value of 0.972 [21].

The ICC values used to measure test–retest reliability for BPII 2.0 and NPI were excellent (0.903 and 0.915, respectively). For BPII 2.0, ICC values ranged from 0.89 and 0.98 in the Indonesian translation, 0.89 in the German translation, 0.97 in the Swedish translation, 0.87 in the Norwegian translation and 0.97 in the original study. [9, 18, 22–24]. The fact that there are 7–10 days between the two questionnaires and that there is no change in the knee conditions of the patients in this interval may indicate the accuracy of the high value obtained by the literature. We did not encounter floor or ceiling effects in our study. Floor and ceiling effects were not seen in the original work of BPII 2.0 and the translation studies into other languages [18, 19, 21]. While there was no floor or ceiling effect for NPI in our study, it was seen that there was a floor effect in the author's responsiveness article that included patients that had first-time patella dislocation and in the Dutch translation [21, 31]. This difference may be since the patients who took the questionnaire in the original study were patients who experienced patella dislocation for the first time; our study included patients who were operated on. In the Dutch translation article, patients between 5–15 years postoperatively were included in the study. In our study, this period was 2–6 years. In both studies, it was suggested to evaluate the floor effect in future studies, and we hope that this study can contribute to the literature on this subject.

Correlation coefficients were determined by comparing the Turkish versions of BPII 2.0 and NPI with the previously translated Kujala and Lysholm scores to evaluate the validity. A very high correlation (*r* = 0.807, *p* < 0.05) was observed between BPII 2.0 and Lysholm score, while a lower correlation was obtained with the Kujala knee score (*r* = 0.529, *p* < 0.05). Although similar results were obtained in the BPII's original author's article, which contains a questionnaire with 32 questions (*r* = 0.50; *p* < 0.001), and the German translation article (*r* = 0.58 *p* < 0.01), high results were obtained in the Indonesian (*r* = 0.98) and Dutch translation (*r* = 0.83) studies [19, 21, 22, 32]. An excellent correlation was observed between the Turkish translation of NPI and Lysholm and Kujala questionnaires in our study (*r* = −0.662 *p* < 0.05 and *r* = −0.819, *p* < 0.05, respectively). In the original study of NPI, while the correlation coefficient between Kujala was *r* = −0.66, this value was *r* = −0.54 for the Lysholm score [16]. The correlation was *r* = 0.63 for Lysholm knee score and NPI in the author's other study, which included patients with patellar dislocation for the first time [31]. In the Brazilian Portuguese translation study, these values were = −0.57 for the Kujala score and *r* = −0.56 for the Lysholm score [25]. In the Dutch translation of NPI, the correlation with Kujala was found to be *r* = −0.78, similar to our study. In the study of Hiemstra et al., in which a 32-question questionnaire was used, the correlation between BPII 2.0 and NPI was found to be *r* = −0.53, a very similar result [32].

There were some limitations in this study. The inclusion of only operated patients in the study also prevented possible floor or ceiling effects. A study with a cohort of operated and non-operated patients with patellofemoral instability may be well reassessed later. The lack of translation and cultural adaptation studies of these two questionnaires to other languages may have left our study incomplete in comparing it with the literature.

## Conclusion

The BPII 2.0 and NPI scores have been successfully translated and adapted to the Turkish population. The findings of this study indicate that the Turkish version of the BPII 2.0 and NPI scores is reliable and valid patient-reported outcome measures of PF instability and can be used to evaluate treatment results for the Turkish population.

### Supplementary Information

**Additional file 1:** Banff Patellofemoral İnstabilite Enstrumani 2.0.

**Additional file 2:** Norwich Patellar İnstabilite Skoru.

## Data Availability

The data used and/or analyzed during the current study are available from the corresponding author on reasonable request.
