# Supplementary material for: Translation, reliability and validity of the Turkish versions of Norwich Patellar Instability score and The Banff Patellar Instability Instrument 2.0
Source: J Orthop Surg Res. 2024 Feb 14;19:140. doi: 10.1186/s13018-024-04612-3 (PMC10865514; doi:10.1186/s13018-024-04612-3)
Supplement: Supplementary file 1 — Additional file 1: Banff Patellofemoral İnstabilite Enstrumani 2.0. [file 13018_2024_4612_MOESM1_ESM.docx]

**BANFF PATELLOFEMORAL İNSTABİLİTE ENSTRÜMANI 2.0**

**Hasta Adı**:

**Muayene Tarihi** (Gün/Ay/Yıl):

**Hangi diz?**

- **Sağ diz**
- **Sol diz**
- **Her iki diz**

**Yönergeler-Talimatlar**

**Lütfen her soruyu sağlam olmayan diz kapağına sahip diziniz ile ilgili mevcut durum, işlev, şartlar ve fikirlerinize göre cevaplayınız. Son 3 ayı göz önünde bulundurunuz.**

**0 ile 100 aralığında uzanan çizgi üzerinde durumunuzu en iyi gösteren noktayı eğik bir çizgi ile işaretleyiniz.**

**Örnek olarak, aşağıdaki soru:**

**Bu iyi bir anket mi?**

**0 100**

**Gereksiz Mükemmel**

**Eğer eğik çizgi, çizginin ortasındaysa, anketin ortalama kalitede olduğunu, bir başka ifadeyle, “gereksiz” ve “mükemmel” uçlarının arasında olduğunu gösterir. Önemli bir nokta olarak: Eğer en uçtaki açıklama durumunuzu tam olarak yansıtıyorsa, eğik çizginizi satırın uçlarına da koyabilirsiniz. Eğer en uç açıklamalar durumunuzu doğru şekilde ifade ediyorsa, eğik çizgiyi satırın iki ucundan birisine koymanız önemlidir.**

**A Bölümü: Semptomlar ve fiziksel şikayetler**

1. Diz kapağınızın yerinden çıkması veya dengesizliği sizi ne kadar rahatsız etmektedir?

0 100

Aşırı derecede rahatsız etmekte Hiç rahatsız etmemekte

1. Uzamış aktivitelerde (yarım saatten daha uzun olan) dizinizde ne kadar ağrı veya rahatsızlık olmaktadır? Örneğin: Ayakta durma, yürüme, spor yapma, vb.

0 100

Şiddetli ağrı Hiç ağrı yok

1. Uzamış oturma durumunda (yarım saatten daha uzun olan) dizinizde ne kadar ağrı veya rahatsızlık olmaktadır? Örneğin: Film izlerken, araba kullanırken, vb.

0 100

Şiddetli ağrı Hiç ağrı yok

1. Dizinizde hareket kaybı var mı?

0 100

Şiddetli hareket kaybı Hareket kaybı yok

1. Dizinizi ne kadar güçsüz hissediyorsunuz?

0 100

Son derece güçsüz Hiç güçsüz değil

**B Bölümü: İş ve/veya okul ile ilgili endişeler**

**Eğer diziniz yüzünden çalışamıyorsanız, her sorudaki çizginin en sol sınırına eğik çizgi işareti koyunuz.

1. İşte ve/veya okulda yön değiştirme ve eksen etrafında dönme hareketleri yaparken diziniz sebebiyle ne kadar zorluk çekiyorsunuz?

0 100

Şiddetli zorluk çekiyorum Hiç zorluk çekmiyorum

1. İşte ve/veya okulda çömelme hareketi yaparken ne kadar zorluk çekiyorsunuz?

0 100

Şiddetli zorluk çekiyorum Hiç zorluk çekmiyorum

1. İşte ve/veya okulda dizinizdeki sorun sebebiyle zaman kaybetmek sizi ne kadar endişelendiriyor?

0 100

Son derece endişelendiriyor Hiç endişelendirmiyor

1. Dizinizdeki sakatlık sebebiyle yapılan harcamalar sizin veya ailenizin bütçesinde zorluk yarattı mı?

0 100

Bütçede şiddetli zorluk yarattı Bütçede hiç zorluk yaratmadı

**Bölüm C: Eğlence, Spor ve Aktiviteler**

1. Eğlence ve/veya spor aktivitelerinizin dizinizi daha kötü yapacağından ne kadar endişeleniyorsunuz?

0 100

Son derece endişelendiriyor Hiç endişelendirmiyor

1. Eğlence ve/veya spor aktivitelerinize katılırken önlem almak zorunda kalıyor musunuz?

(Eğer eğlence ve/veya spor aktivitelerinize diziniz yüzünden katılamıyorsanız çizginin en soluna (0) eğik çizgi koyunuz)

0 100

Her zaman önlem alıyorum Hiç önlem almıyorum

1. Eğlence ve/veya spor aktiviteleri yaparken diz kapağınızın yerinden çıkmasından ne kadar korkuyorsunuz?

(Eğer eğlence ve/veya spor aktivitelerinize diziniz yüzünden katılamıyorsanız çizginin en soluna (0) eğik çizgi koyunuz)

0 100

Son derece korkutuyor Hiç korkutmuyor

1. Engebeli arazide, ıslak zeminde veya buz üzerinde yürüme sizi ne kadar endişelendiriyor?

0 100

Son derece endişelendiriyor Hiç endişelendirmiyor

1. Eğlence ve/veya spor aktivitelerinde tam eforunuzu sarf edebiliyor musunuz?

(Eğer eğlence ve/veya spor aktivitelerinize diziniz yüzünden katılamıyorsanız çizginin en soluna (0) eğik çizgi koyunuz).

0 100

Hiç efor sarf edemiyorum Her zaman tam efor sarf edebiliyorum

**D Bölümü: Hayat Tarzı**

1. Dizinizdeki problem sebebiyle oluşan genel güvenlik sorunları sizi ne kadar endişelendiriyor? Örneğin: Merdivenden aşağı inme veya yukarı çıkma, araba sürme veya küçük çocukları taşıma, vb.

0 100

Son derece endişelendiriyor Hiç endişelendirmiyor

1. Dizinizdeki problem sebebiyle, egzersiz yapma ve formda kalma kabiliyetinizin ne kadarı kısıtlandı?

0 100

Tamamen kısıtlandı Hiç kısıtlanmadı

1. Dizinizdeki problem yüzünden hayattan aldığınız keyif ne kadar kısıtlandı?

0 100

Tamamen kısıtlandı Hiç kısıtlanmadı

1. Dizinizdeki problem sebebiyle ailenizle ve/veya arkadaşlarınızla yaptığınız hayat tarzı aktivitelerinden kaçınıyor musunuz?

0 100

Her zaman kaçınıyorum Hiç kaçınmıyorum

1. Dizinizdeki problem yüzünden hayat tarzı veya sosyal aktiviteleriniz hakkında ailenizden ve/veya arkadaşlarınızdan daha fazla planlama yapmanız gerekiyor mu?

0 100

Her zaman planlama yapmak zorundayım Hiç planlama yapmak zorunda değilim

**Bölüm E: Sosyal ve Duygusal**

1. Diz probleminiz nedeniyle eğlence veya rekabetçi ihtiyaçlarınızın artık karşılanamamasından dolayı hayal kırıklığı yaşıyor musunuz?

(Eğer rekabetçi ihtiyaçlarınız karşılanıyorsa çizginin en sağına (100) eğik çizgi koyunuz. Eğer hiç rekabetçi ihtiyacınız yoksa çizginin en soluna (0) eğik çizgi koyunuz)

0 100

Son derece yaşıyorum Hiç yaşamıyorum

1. Dizinizdeki problem ile duygusal olarak başa çıkma konusunda hiç zorluk yaşadınız mı?

0 100

Şiddetli zorluk yaşadım Hiç zorluk yaşamadım

1. Diziniz ne sıklıkla sinirinizi bozuyor?

0 100

Her zaman Hiçbir zaman

1. Dizinizi tekrar sakatlamaktan ne kadar korkuyorsunuz?

0 100

Son derece korkuyorum Hiç korkmuyorum

**Anketi tamamladığınız için teşekkür ederiz**
