# Supplementary material for: Translation, reliability and validity of the Turkish versions of Norwich Patellar Instability score and The Banff Patellar Instability Instrument 2.0
Source: J Orthop Surg Res. 2024 Feb 14;19:140. doi: 10.1186/s13018-024-04612-3 (PMC10865514; doi:10.1186/s13018-024-04612-3)
Supplement: Supplementary file 2 — Additional file 2: Norwich Patellar İnstabilite Skoru. [file 13018_2024_4612_MOESM2_ESM.docx]

**Norwich Patellar İnstabilite Skoru**

Aşağıda diz kapağınızı “yerinden çıkacak” gibi veya dengesiz hissettirebilecek bir dizi aktivite yer almaktadır.

Lütfen aşağıdaki bütün ifadeleri okuyup, belirtilen aktiviteleri yaptığınızda hangi sıklıkta diz kapağınızın “yerinden çıkacak” gibi olduğunu veya dengesiz hissettirdiğini en iyi tanımlayan kutucuğu işaretleyiniz.

Lütfen bütün sorular için bir kutucuğu işaretleyin

| # | Soru | Her zaman | Sık sık | Bazen | Nadiren | Hiçbir zaman | Yapmı- yorum |
| --- | --- | --- | --- | --- | --- | --- | --- |
| 1 | Spor/oyun esnasında dönme, yön değiştirme |  |  |  |  |  |  |
| 2 | Koşarken yön değiştirme |  |  |  |  |  |  |
| 3 | Engebeli zeminde düz bir çizgide koşma |  |  |  |  |  |  |
| 4 | Kaygan, ıslak veya buzlu bir zeminde yürüme |  |  |  |  |  |  |
| 5 | Yana doğru koşma |  |  |  |  |  |  |
| 6 | Hoplama |  |  |  |  |  |  |
| 7 | Zıplama |  |  |  |  |  |  |
| 8 | Düz zeminde düz bir çizgide koşma |  |  |  |  |  |  |
| 9 | Merdivenden inme |  |  |  |  |  |  |
| 10 | Çömelme |  |  |  |  |  |  |
| 11 | Diz üzerine oturma |  |  |  |  |  |  |
| 12 | Engebeli zeminde düz bir çizgide yürüme |  |  |  |  |  |  |
| 13 | Merdiven çıkma |  |  |  |  |  |  |
| 14 | Yüksek basamağa çıkma veya üstünden geçme |  |  |  |  |  |  |
| 15 | Otururken bacak bacak üstüne atmak |  |  |  |  |  |  |
| 16 | Düz zeminde düz bir çizgide yürüme |  |  |  |  |  |  |
| 17 | Arabaya binme veya arabadan inme |  |  |  |  |  |  |
| 18 | Market arabasını reyonlar arasında döndürme |  |  |  |  |  |  |
| 19 | Arkaya bakmak için dönme |  |  |  |  |  |  |
